# Stereospecific Epoxidation of Limonene Catalyzed by Peroxygenase from Oat Seeds

**DOI:** 10.3390/antiox10091462

**Published:** 2021-09-14

**Authors:** Daniela Maria Biondi, Claudia Sanfilippo, Angela Patti

**Affiliations:** Istituto di Chimica Biomolecolare, CNR, Via Paolo Gaifami 18, I-95126 Catania, Italy; daniela.biondi@icb.cnr.it (D.M.B.); claudia.sanfilippo@cnr.it (C.S.)

**Keywords:** limonene, biocatalysis, peroxygenase, epoxidation, oat flour

## Abstract

Limonene is one of the most abundant naturally occurring cyclic monoterpenes and has recently emerged as a sustainable alternative to petroleum-based solvents as well as a chemical platform for the production of value-added compounds. The biocatalytic epoxidation of both enantiomers of limonene was carried out in the presence of a peroxygenase-containing preparation from oat (*Avena sativa*) flour. Different reaction profiles were observed depending on the starting enantiomer of limonene, but in both cases the 1,2-monoepoxide was obtained as the main product with excellent diastereoselectivity. *Trans*-1,2-monoepoxide and *cis*-1,2-monoepoxide were isolated from the reaction of (*R*)-limonene and (*S*)-limonene, respectively, and the reactions were scaled-up to 0.17 M substrate concentration. The process is valuable for operational simplicity, lack of toxic metal catalysts, and cost-effectiveness of the enzymatic source. Pure stereoisomers of 1,2-monoepoxides of limonene constitute a useful starting material for biorenewable polymers, but can be also converted into other chiral derivatives by epoxide ring opening with nucleophiles. As a proof of concept, a tandem protocol for the preparation of enantiopure (1*S*,2*S*,4*R*)-1,2-diol from (*R*)-limonene and (1*R*,2*R*,4*S*)-1,2-diol from (*S*)-limonene was developed.

## 1. Introduction

Limonene is one of the most abundant naturally occurring cyclic monoterpenes and its (*R*)-(+)-enantiomer is easily obtained by extraction from citrus oil, wherein it is present in more than 90%, whereas the antipode (*S*)-(−)-limonene is one of the major components of oaks and pines essential oils.

Beside the traditional use as flavor additive for fragrance and food industries [1], limonene provides a green and sustainable alternative to petroleum-based solvents in chemical extractions [2] and chromatography [3], in surfactant formulations [4], and for cleaning purposes [5]. For its antimicrobial and biological activities, limonene is used as insecticide and as dietary supplement in nutraceutical formulations [6,7]. Furthermore, great potential for medical uses comes from the interesting anticancer and cancer pre-vention properties exhibited by this monoterpene [8,9].

Currently, growing interest is also focused on the use of limonene as a chemical platform [10] for the production of value-added compounds and, in this context, many efforts have been directed to the development of bio-based polymers [11,12,13] for specialized applications and with enhanced biodegradability.

Epoxidation of one or both double bonds of limonene opens the way for further functionalization and limonene 1,2-epoxide is the starting material for the production of limonene polycarbonate following its polymerization with carbon dioxide [14,15]. Clean epoxidation of limonene has been extensively investigated with different oxidants in the presence of heterogeneous metal catalysts [16,17,18,19,20], as well as in the presence of lipase for the in-situ generation of a peracid oxidant [21,22]. Direct diastereomeric separation of the *cis*/*trans*-mixture of (+)-limonene oxide has been achieved by exploiting the selective epoxide ring opening of one of the diastereomers with different nucleophiles [23,24,25,26,27] or by using epoxide hydrolases with complementary stereoselectivity [28]. Stereoselective methods based on salen-type catalysts [29,30] or whole-cell microbiological modification of limonene [31] have also been reported, but the target products been obtained in moderate optical purity or in mixture with other related compounds.

Oxygenases (E.C. 1.13, monooxygenases and E.C. 1.14, dioxygenases) could be the ideal biocatalysts since they use molecular oxygen as oxidant. However, when enzymes of the cytochrome P450 monooxygenases family were applied to the epoxidation of limonene, only carveol and perillyl alcohol were obtained [32]. Instead, haloperoxidases and peroxygenases (EC 1.11.2), which both contain a heme-oxoferryl prostetic group and are cofactor-independent, were shown to be active catalysts for the selective oxyfunctionalization of aromatic and aliphatic compounds [33,34] as well as for the epoxidation of limonene with H_2_O_2_ [35,36]. In the presence of chloroperoxidase from *Caldariomyces fumago* (CPO), limonene was regio- and diastereoselectively transformed into the 1,2-epoxide which, however, suffered hydrolytic opening in the reaction conditions to give the corresponding diol [33]. More recently, a recombinant plant peroxygenase from *Solanum lycopersicum* has been reported to promote the selective formation of limonene 1,2-epoxide with different oxidants, albeit in low yield [37].

In our previous work, we reported that the freeze-dried aqueous extract of defatted flour from oat seed (*Avena sativa*) exhibits peroxygenase activity, being able to catalyze the regio- and stereoselective epoxidation of eicosapentaenoic acid (EPA, 20:5 ω-3) using *tert*-butyl hydroperoxide (*t*-BuOOH) as oxygen source [38]. Prompted by this result and the easy availability of the enzyme source, we planned to test the effectiveness of our enzymatic preparation in the epoxidation of limonene, also with the aim to explore and extend the scope of this biocatalytic reaction.

## 2. Materials and Methods

### 2.1. General

(*R*)-(+)-limonene (>99%) and (*S*)-(−)-limonene (95%) were obtained from Tokyo Chemical Industries (Tokyo, Japan). Lyophilized enzymatic preparation from oat seed flour was obtained as previously reported [38] and its activity in the epoxidation of oleic acid was 0.7 μmol/mg/h. TLC (Thin layer chromatography) analyses were performed on aluminium plates coated with silica gel and fluorescent indicator F254, revealing the compounds by UV (Ultraviolet) and cerium sulphate solution. Column chromatography was performed on silica gel (40–63 µm, Merck, Kenilworth, NJ, USA) using the specified eluents. Deactivated silica was prepared by suspending silica (50 g) in *n*-hexane (100 mL) containing 1% (*v*/*v*) triethylamine under stirring for 15 min. Then, the solid was collected by decantation, rinsed with *n*-hexane, and dried at 40 °C overnight.

^1^H- and ^13^C-NMR (Nuclear Magnetic Resonance) spectra were recorded on a Bruker Avance^TM^ 400 spectrometer (Bruker Milano, Italy) at 400.13 and 100.62 MHz, respectively. Chemical shifts (δ) are given as ppm relative to the residual solvent peak and coupling constants (*J*) are in Hz.

Optical rotations were measured on DIP-135 polarimeter (Jasco Europe, Lecco, Italy) using a 10 cm length cell.

### 2.2. GC (Gas-Chromatography) Analysis

GC analyses were performed on a fast GC Shimadzu 17-A instrument (Shimadzu, Milano, Italy), equipped with a flame ionization detector and a Supelco SPB-5 capillary column (15 m × 0.1 mm ID (Inner diameter) × 0.1 μm film thickness, Supelco, Bellafonte, PA, USA). For the analyses, the following parameters were set: He flow rate 0.9 mL/min, split ratio 1:247, injector temperature 250 °C, detector temperature 280 °C. The oven temperature was held at 60 °C for 1 min, then raised to 280 °C at 10 °C/min.

The same chromatographic conditions were also applied for GC-MS analyses, which were carried out on a fast GC Shimadzu 17-A instrument equipped with MS-EI detector (GCMS-QP5050A, Shimadzu, Milano, Italy) using He flow rate 0.9 mL/min and split ratio 1:54. MS-EI (Mass Spectrometry-Electron Impact) detection was carried out with 70 eV ionization voltage, 900 V electron multiplier voltage, and 180 °C ion source temperature. Mass spectra data were acquired in the scan mode in *m*/*z* range of 40–400 and compared with those in NIST (National Institute of Standards and Technology) mass spectral library.

### 2.3. General Procedure for Biocatalyzed Epoxidation of (R)- or (S)-Limonene

(*R*)-limonene or (*S*)-limonene (18 µL, 15 mg, 0.11 mmol) was added to a suspension of peroxygenase-containing preparation from oat flour (100 mg) in 50 mM phosphate buffer at pH 7.5 (7 mL or 5 and 3 mL for study on different substrate concentration) and *t*-BuOOH (70% solution in water, 15 µL, 0.11 mmol) was added in two aliquots over 1 h. The reaction mixture was vigorously stirred at 25 °C and the reaction progress monitored by GC. To aliquots (0.4 mL) of the reaction mixture, Et_2_O (0.4 mL) was added in an Eppendorf vial and the suspension vortexed for 30 s. After centrifugation, the organic phase was separated as the upper layer, collected, and directly used for GC analysis.

### 2.4. Preparative Biocatalyzed Epoxidation of (R)-Limonene

To a suspension of peroxygenase-containing preparation from oat flour (3 g) in 50 mM phosphate buffer (20 mL) at pH 7.5, (*R*)-limonene (540 µL, 454 mg, 3.33 mmol), *t*-BuOOH (70% solution in water, 460 µL, 3.36 mmol) was added at constant infusion rate of 300 µL/h with a syringe pump, maintaining the mixture under vigorous stirring at 25 °C. After 4 h the GC profile of the reaction mixture showed unreacted limonene (9%), monoepoxide **2** (76%) and carveol **3** (11%) as main constituents. The whole suspension was then extracted with Et_2_O (3 × 15 mL) separating each time the organic phase by centrifugation at 4000 RPM (Revolutions per minute) (2930× *g*) for 20 min. The pooled organic phases were dried over anhydrous Na_2_SO_4_ and the solvent was removed by rotavapor at 25 °C and 650 mbar. The residue was purified by column chromatography on deactivated silica gel, eluting with *n*-hexane: Et_2_O 96:4 (*v*/*v*) to give (*R*)-limonene epoxide **2** (340 mg, 2.23 mmol, 67% yield, >98% chemical purity by GC) as a clear oil, [α]_D_^25^ = +70.7 (*c* 0.37, CHCl_3_), lit. [24] [α]_D_ = +76 (*c* 0.98, CHCl_3_). ^1^H- and ^13^C-NMR spectra were in agreement with reported data [39].

Further elution of chromatographic column with *n*-hexane: Et_2_O 94:6 (*v*/*v*) allowed to isolate *trans*-carveol **3** (45 mg, 0.30 mmol, 9% yield, 98% chemical purity by GC), [α]_D_^25^ = +139.5 (*c* 0.85, CHCl_3_), lit. [40] [α]_D_ = +139.6 (*c* 0.5, CHCl_3_), whose NMR spectra were in agreement with reported data [40].

### 2.5. Preparative Biocatalyzed Epoxidation of (S)-Limonene

(*S*)-Limonene (540 µL, 454 mg, 3.33 mmol) was left to react in the presence of peroxygenase-containing preparation from oat flour and *t*-BuOOH as described above. After 3 h, the GC profile of the reaction mixture showed unreacted limonene (6%), monoepoxide **4** (68%), and diepoxide **5** (14%) as main constituents. The work-up as above gave a residue that was purified by column chromatography on deactivated silica gel, eluting with *n*-hexane: Et_2_O 96:4 (*v*/*v*) to give (*S)*-limonene epoxide **4** (300 mg, 1.97 mmol, 59% yield, >98% chemical purity by GC) as a clear oil, [α]_D_^25^ = −42.9 (*c* 0.92, CHCl_3_), lit. [26] [α]_D_ = −38.0 (*c* 3.5, CHCl_3_). ^1^H- and ^13^C-NMR spectra were in agreement with reported data [39].

Further elution of chromatographic column with *n*-hexane: Et_2_O 85:15 (*v*/*v*) gave diepoxide **5** (65 mg, 0.39 mmol, 12% yield, 96% chemical purity by GC), [α]_D_^25^ = −38.1 (*c* 0.85, CHCl_3_), ^1^H-NMR (CDCl_3_): δ 1.03 (m, 1H, H-4), 1.23 (s, 3H, H-10), 1.30 (s, 3H, H-7), 1.52 (m, 3H, H-3a, H-5a, H-6a), 1.86 (m, 2H, H-5b, H-6b), 2.17 (m, 1H, H-3b), 2.50 and 2.61 (d, 1H each, AB system, *J* = 4.8 Hz); ^13^C-NMR (CDCl_3_): δ 18.7 (C-10), 23.5 (C-5), 24.3 (C-7), 27.7 (C-3), 28.7 (C-6), 34.8 (C-4), 52.6 (C-9), 57.26 (C-1), 58.7 (C-8), 60.4 (C-2).

### 2.6. Synthesis of Diepoxide (−)-***5*** from Monoepoxide (−)-***4***

To a suspension of peroxygenase preparation from oat (500 mg) and monoepoxide (−)-**4** from (*S*)-limonene (50 mg, 0.33 mmol) in 50 mM phosphate buffer (15 mL) at pH 7.5, *t*-BuOOH (70% solution in water, 48 µL, 0.35 mmol) was added in two aliquots over 1 h and the suspension maintained under stirring at 25 °C. The starting substrate fully reacted in 4 h and the mixture was then extracted with Et_2_O. After work-up as above, diepoxide (−)-**5** was obtained in 87% isolated yield (48 mg, 0.29 mmol).

### 2.7. Synthesis of Diols (+)-***6*** and (−)-***7*** from (R)-Limonene

(*R*)-limonene (200 μL, 168 mg, 1.23 mmol) was added to 50 mM phosphate buffer at pH 7.4 (7 mL) containing lyophilized extract from oat flour (1 g). To this suspension *t*-BuOOH (70% solution in water, 170 μL, 1.24 mmol) was added at constant infusion rate of 100 μL/h using a syringe pump. The reaction was stirred vigorously at 25 °C and the substrate conversion was monitored by GC analysis. After 4 h, when the substrate conversion reached 90% (76% of monoepoxide **2**), the reaction was stopped, and the pH adjusted to 3.5 with H_3_PO_4_. The mixture was maintained at room temperature under magnetic stirring until the complete disappearance of the limonene oxide (3 h) was evidenced by TLC analysis (*n*-hexane/EtOAc 60:40 *v*/*v*). The reaction was extracted with EtOAc (5 mL × 3) and the collected organic layers were dried over anhydrous Na_2_SO_4_. After evaporation of the solvent at reduced pressure, the residue was purified on silica gel column eluting with *n*-hexane/EtOAc 70:30 *v/v* to give (+)-**6** (100 mg, 0.59 mmol, 48% yield, 98% chemical purity by GC, mp 73 °C) and (−)-**7** (33 mg, 0.19 mmol, 16% yield 98% chemical purity by GC, mp 74 °C) as white solids (84% global yield of the two diols with respect to the starting monoepoxide). Physical properties and NMR spectra were in agreement with those reported in the literature [41]. For (+)-(1*S*,2*S*,4*R*)-**6**: [α]_D_^25^ = +25.3 (*c* 2.7, CHCl_3_), lit. [41] [α]_D_^25^ = +25.8 (*c* 1.0, CHCl_3_); for (−)-(1*R*,2*R*,4*R*)-**7**: [α]_D_^25^ = −4.8 (*c* 1.0, CHCl_3_), lit. [39] [α]_D_^25^ = −5.0 (*c* 1.0, CHCl_3_).

### 2.8. Synthesis of Diol (−)-***6*** from (S)-Limonene

(S)-Limonene (200 μL, 168 mg, 1.23 mmol) was added to 50 mM phosphate buffer at pH 7.4 (7 mL) containing lyophilized extract from oat flour (1 g). To this suspension, *t*-BuOOH (70% solution in water, 170 μL, 1.24 mmol) was added at constant infusion rate of 100 μL/h using a syringe pump. After 3 h the substrate conversion reached 92% (72% of monoepoxide **4**) and the pH was adjusted to 3.5 with H_3_PO_4_. Complete conversion of **4** to **6** was detected by TLC after 1 h. Following the same work-up as above, diol (−)-(1*R*,2*R*,4*S*)-**6** was isolated (130 mg, 0.77 mmol, 63% yield, >98% chemical purity by GC) in 87% yield with respect to the starting monoepoxide. [α]_D_^25^ = −25.1 (*c* 2.3, CHCl_3_), lit. [41] [α]_D_^25^ = +25.8 (*c* 1.0, CHCl_3_) for its enantiomer.

## 3. Results

The biocatalyzed epoxidation of (*R*)-limonene, (*R*)-**1**, was carried out using a freeze-dried extract of flour from oat seeds as peroxygenase source and *t*-BuOOH as oxidant. As previously reported [38], the peroxygenase-containing preparation was obtained by the aqueous extraction of flour of oat seed followed by low gravity centrifugation to maintain the microsomal fraction as a suspension. Freeze-drying of this suspension was crucial to preserve the activity of the enzyme up to six months at −20 °C storage and ensure reproducibility within the same batch of enzyme preparation.

**Scheme 1:**
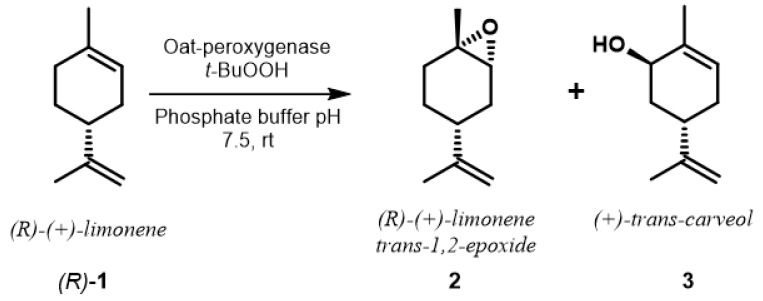
Peroxygenase catalyzed epoxidation of (*R*)-limonene.

A first reaction was carried out using a (*R*)-limonene:*t*-BuOOH 1:2 molar ratio in phosphate buffer at pH 7.5 and the progress of the biocatalytic reaction was followed by GC-MS analysis. About 85% conversion of (*R*)-**1** was reached in 20 h and limonene 1,2-oxide **2** was present as the main product (72%) in the reaction mixture together with carveol **3** (10%) and a few other minor components (Scheme 1). The formed monoepoxide **2** was present as a single diastereoisomer (*dr* 99:1) and it was identified as the *trans*-isomer by GC co-injection with pure standard. (A typical GC-profile of biocatalyzed epoxidation of (*R*)-limonene is shown in Figure S1 of Supplementary Material). Epoxide **2** was found stable in our reaction conditions and diols deriving from the hydrolytic opening of the oxirane ring were not detected.

In a parallel reaction carried out by adding the oxidant in two portions, a marked increase of the reaction rate was observed, and a comparable composition of the final reaction mixture was obtained within 2 h. This evidence suggested the occurrence of some enzyme inactivation by *t*-BuOOH, mitigated by decreasing its local concentration over the time. Limonene diepoxide was not detected, even at longer reaction times, indicating high regioselectivity of the enzyme toward the *endo*-cyclic double bond compared to the *exo*-cyclic one.

A subsequent reaction was then carried out by using *t*-BuOOH in 1:1 molar ratio with the substrate and, in these conditions, complete conversion of the substrate (99%) was obtained just in 2 h, with slightly increased yields of **2** (79%) and **3** (15%).

Interestingly, the carveol by-product **3** was exclusively obtained as the *trans*-isomer suggesting that its formation results from an enzyme-catalyzed process rather than the autoxidation of (*R*)-**1** [42]. Considering that hydroxylation reactions have also been reported for peroxygenases, we carried out a qualitative test for this activity [43] and it was found that indole is converted into indoxyl in the presence of our enzymatic preparation. 

The observed formation of carveol could hence result from a peroxygenase-promoted abstraction of the cyclic allylic hydrogen and subsequent oxidation in this position, in analogy with the regiospecific 6-hydroxylation of (+)-limonene to (+)-*trans*-carveol previously reported to occur in the presence of microsomial preparations containing cytochrome P-450 from fruits [44] or human liver [32], as well as with whole cells from different bacterial strains [45,46] as biocatalysts.

**Scheme 2:**
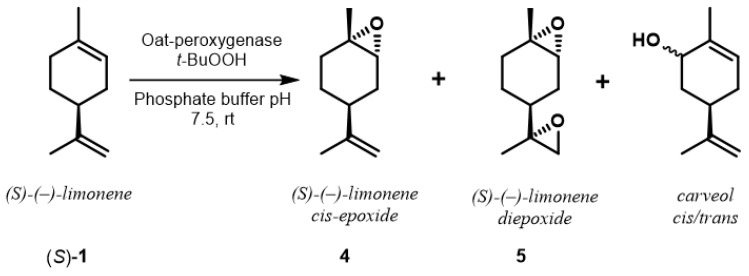
Peroxygenase catalyzed epoxidation of (*S*)-limonene.

The optimized reaction conditions (1:1 substrate:*t*-BuOOH ratio, oxidant added in two portions) were then applied to the epoxidation of (*S*)-(−)-limonene, (*S*)-**1**, which proceeded with slightly higher rate and comparable diastereoselectivity (*dr* 98:2) with respect to (*R*)-**1**, giving *cis*-1,2-epoxide **4** as main product (83%). In comparison with the reaction on (*R*)-**1**, carveol **3** was detected in both diastereoisomeric forms and in sensibly decreased amounts (3% total), while a diepoxide derivative **5** was present as the major side-product (7%) (Scheme 2). A typical GC-profile of biocatalyzed epoxidation of (*S*)-limonene is shown in Figure S2 of Supplementary Material).

The formation of **4** supports the occurrence of a selective recognition by the enzyme of the same face of the double bond of limonene, regardless of the stereochemistry of the 4-isopropenyl substituent, and this is in agreement with data reported for the reaction catalyzed by another plant peroxygenase from *Solanum lycopersicum* [37], while opposite stereoselectivity was observed with fungal enzymes [35,36].

Compared to other peroxygenase-catalyzed epoxidation reactions on limonene, these preliminary results were excellent in terms of both substrate loading and yield of the target monoepoxides, paving the way to a preparative scale-up. No significant differences were observed in the reaction profiles after increasing the concentration of limonene four-fold with the same substrate: enzyme ratio, and in preparative runs we went up to 0.17 M limonene, while the addition of *t*-BuOOH was diluted over 2 h with a syringe pump. With both (*R*)- and (*S*)-limonene about 90% of substrate conversion was reached and about 70% of the target monoepoxide was present in the reaction mixture. Since it is known that some loss of limonene 1,2- oxide can occur during its purification due to its volatility [27], a careful work-up of the reaction mixture was set-up, using diethyl ether as extraction solvent and controlling pressure during its evaporation. Further improvement in the yields of the isolated monoepoxides was achieved by column chromatography on base-deactivated silica gel.

From a preparative reaction on (*R*)-limonene, *trans*-monoepoxide **2** and carveol **3** were isolated by column chromatography and their structure was confirmed on the basis of their known NMR spectra. Biocatalyzed oxidation of (*S*)-limonene in the same conditions, instead, led to the isolation of *cis*-monoepoxide **4** and diepoxide **5**, whose stereochemistry was assigned as (1*S*,2*R*,4*S*,8*S*)-**5** by comparison of its ^1^H- and ^13^C-NMR resonances with those reported in literature [47] (see Figures S4 and S5 in Supplementary Material). Noteworthy, the other possible diepoxide deriving from **4**, i.e., the diastereoisomeric (1*S*,2*R*,4*S*,8*R*)-**5**, was not detected in the reaction mixture, indicating that the epoxidation on the *exo*-double bond of (*S*)-limonene also proceeds with high stereoselectivity.

Attempts to drive the epoxidation of (*S*)-limonene toward the formation of **5** were carrried out by using doubled amount of *t*-BuOOH oxidant, but the reaction sensibly slowed down after 2 h, when about 40% of **5** was formed. However, starting from dilute solution of **4** (0.02 M), quantitative conversion to **5** was reached in 4 h in the presence of 1:1 substrate:*t*-BuOOH ratio and increased amount of the enzyme (The GC-profile of biocatalyzed epoxidation of (*S*)-limonene-1,2-epoxide at 50% substrate conversion is shown in Figure S3 of Supplementary Material). Under the same conditions, instead, monoepoxide **2** from (*R*)-limonene did not react at all.

Although some protocols have been developed for the *bis*-epoxidation of limonene [48,49,50], the product has been obtained as a mixture of diastereoisomers difficult to separate, and this is the first report of the isolation and characterization of an enantiomerically pure diepoxide of limonene.

All these data show that oat peroxygenase is a multifaceted enzyme able to catalyze (a) the regioselective epoxidation of the *endo*-double bond of both enantiomers of limonene with the same excellent enantiofacial discrimination; (b) the stereoselective 6-hydroxylation of (*R*)-limonene only; (c) the epoxidation of *exo*-double bond of (S)-limonene only.

Since the epoxide ring-opening by nucleophiles occurs with known outcome depending on the starting diastereoisomer of limonene 1,2-epoxide [51], the peroxygenase-catalyzed epoxidation of limonene here developed can be also exploited in a tandem protocol for the preparation of other limonene derivatives. As an example, thanks to the occurrence of a selective axial nucleophilic attack of water on the epoxide ring in mild acidic conditions, which results in the preferential formation of *trans*-diaxial diol **6** [25], both enantiomers of diol **6** could be prepared from (*R*)- and (*S*)-limonene.

**Scheme 3:**
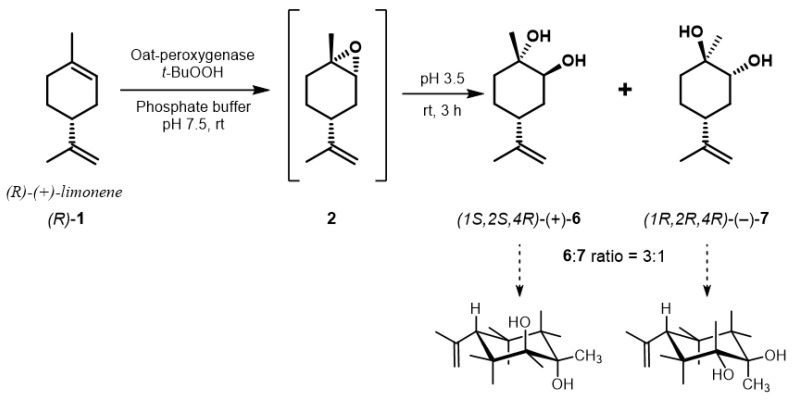
Formation of (*R*)-limonene 1,2-diols by tandem biocatalyzed epoxidation-hydrolytic ring opening.

**Scheme 4:**
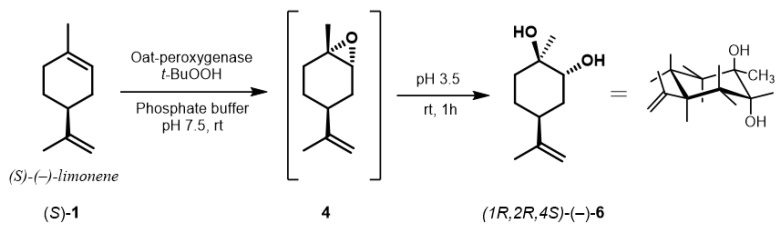
Selective formation of (S)-limonene 1,2-diol by tandem biocatalyzed epoxidation-hydrolytic ring opening.

As a proof of the concept, at the end of biocatalyzed epoxidation the whole reaction mixture was taken to pH 3.5 by addition of dil. H_3_PO_4_ and left to stand at room temperature for 1–3 h. In these conditions, diols (+)-**6** and (−)-**7** in a 3:1 ratio were obtained from (*R*)-limonene (Scheme 3), while (−)-**6** was exclusively formed from (*S*)-limonene (Scheme 4).

## 4. Conclusions

A simple and economic biocatalyzed procedure was developed for the epoxidation of both enantiomers of limonene, using a peroxygenase-containing preparation from oat flour as an enzymatic source. Compared to reactions carried out with purified peroxigenases from different sources, the method provides advantages, including easy and cheap access to the biocatalyst and feasibility on a preparative scale. Peroxygenase from oat displayed stereospecificity in the epoxidation of limonene, giving different products depending on the chirality of the starting substrate. *Trans*-1,2-epoxide from (*R*)-limonene and *cis*-1,2-epoxide from (*S*)-limonene were obtained as single diastereoisomers, allowing further derivatization of these compounds by using tandem protocols. Following this approach, both enantiomers of *trans*-diaxial 1,2-limonene diol were prepared by hydrolytic opening of the epoxide ring in good yields. In addition, enantiopure (+)-*trans*-carveol resulting from a selective 6-hydroxylation of (*R*)-limonene and (1*S*,2*R*,4*S*,8*S*)-*bis*-epoxide from (*S*)-limonene were isolated from the epoxidation reaction mixtures.

## Data Availability

The data presented in this study are available in this manuscript.

## Supplementary Materials

The following are available online at https://www.mdpi.com/article/10.3390/antiox10091462/s1, Figures S1–S3: GC profiles of biocatalyzed epoxidation of (*R*)-limonene, (*S*)-limonene and (S)-limonene-1,2-epoxide, Figures S4 and S5: NMR spectra of (S)-limonene-diepoxide obtained by biocatalyzed epoxidation.
