# Supplementary material for: Stereospecific Epoxidation of Limonene Catalyzed by Peroxygenase from Oat Seeds
Source: Antioxidants (Basel). 2021 Sep 14;10(9):1462. doi: 10.3390/antiox10091462 (PMC8469233; doi:10.3390/antiox10091462)
Supplement: Supplementary file 1 [file antioxidants-10-01462-s001.zip › antioxidants-1371738-supplementary.pdf]

Stereospecific epoxidation of limonene catalyzed by peroxygenase from oat seeds

Daniela Maria Biondi, Claudia Sanfilippo, Angela Patti*

Istituto di Chimica Biomolecolare, CNR – Via Paolo Gaifami 18, I-95126 Catania, Italy

* Correspondence: angela.patti@cnr.it

GC profile of the biocatalyzed epoxidation of (*R*)-limonene	Page	SI-1
GC profile of the biocatalyzed epoxidation of (*S*)-limonene		SI-2
GC profile of the biocatalyzed epoxidation of (*S*)-limonene-1,2-epoxide		SI-3
NMR spectra of (*S*)-limonene-diepoxide obtained by biocatalyzed epoxidation		SI-4

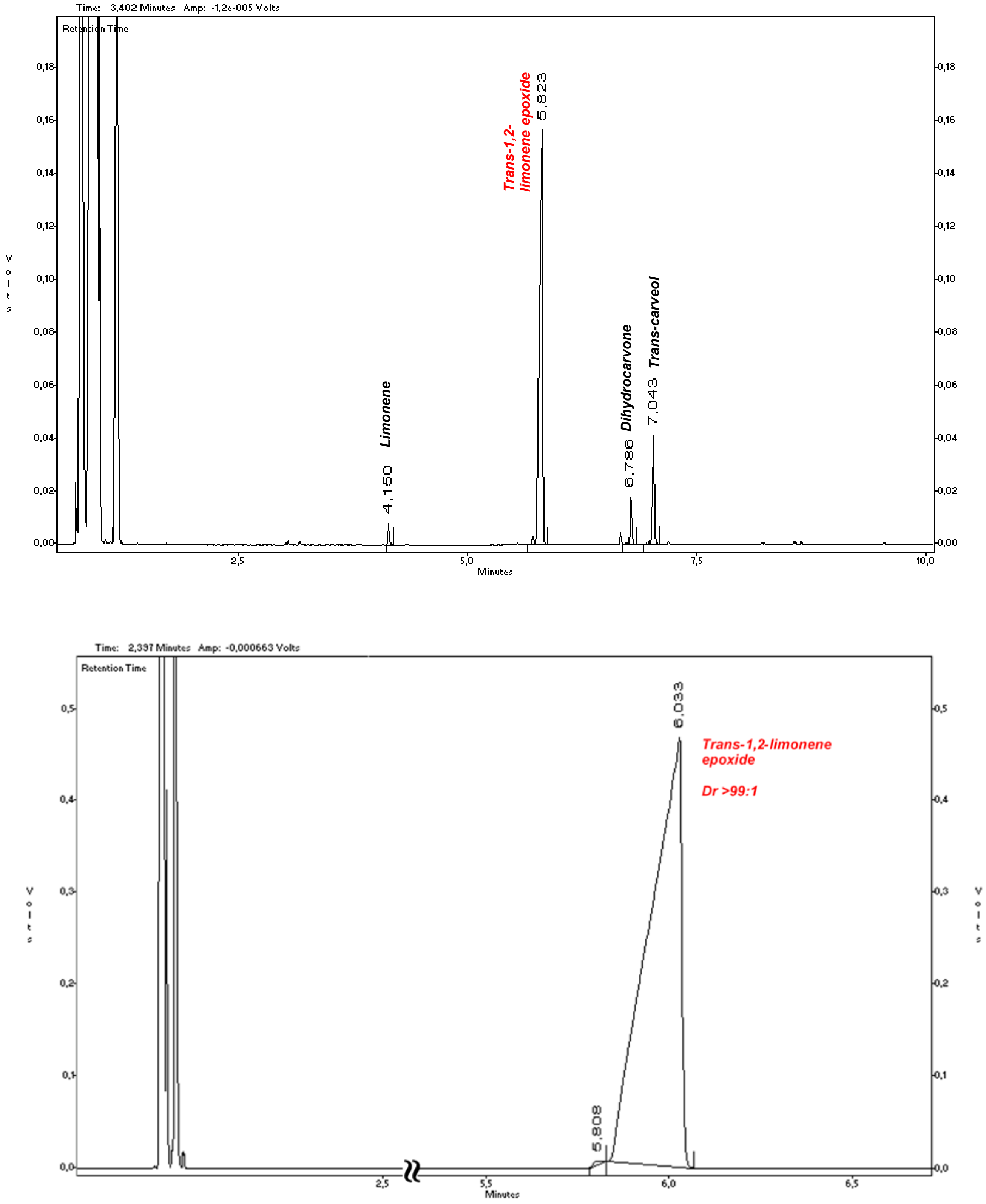

Figure S1. GC profile of the biocatalyzed epoxidation of (*R*)-limonene (top) and diastereoisomeric purity of the isolated (*R*)-limonene-1,2-epoxide (bottom)

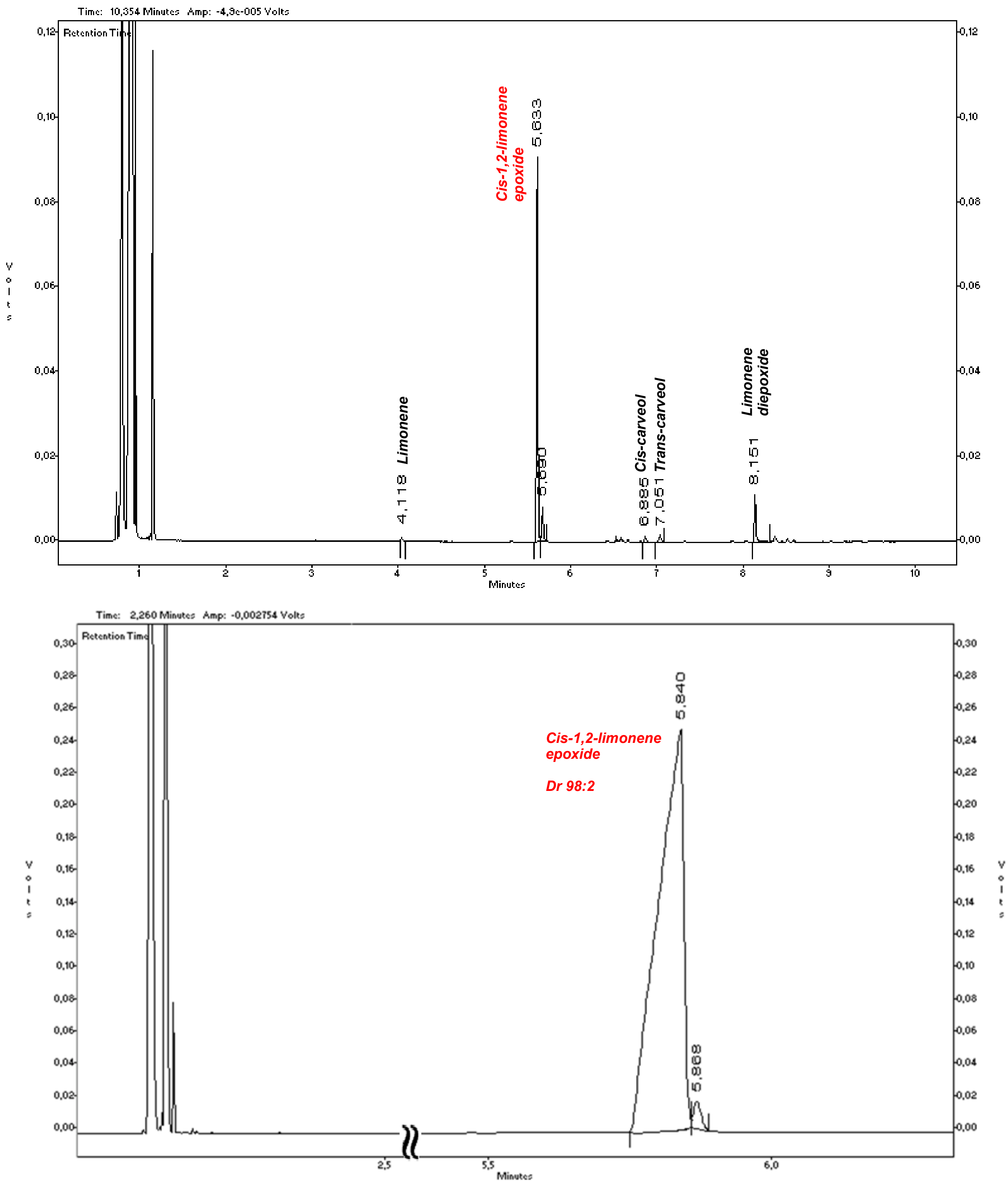

Figure S2. GC profile of the biocatalyzed epoxidation of (*S*)-limonene (top) and diastereoisomeric purity of the isolated (*S*)-limonene-1,2-epoxide (bottom)

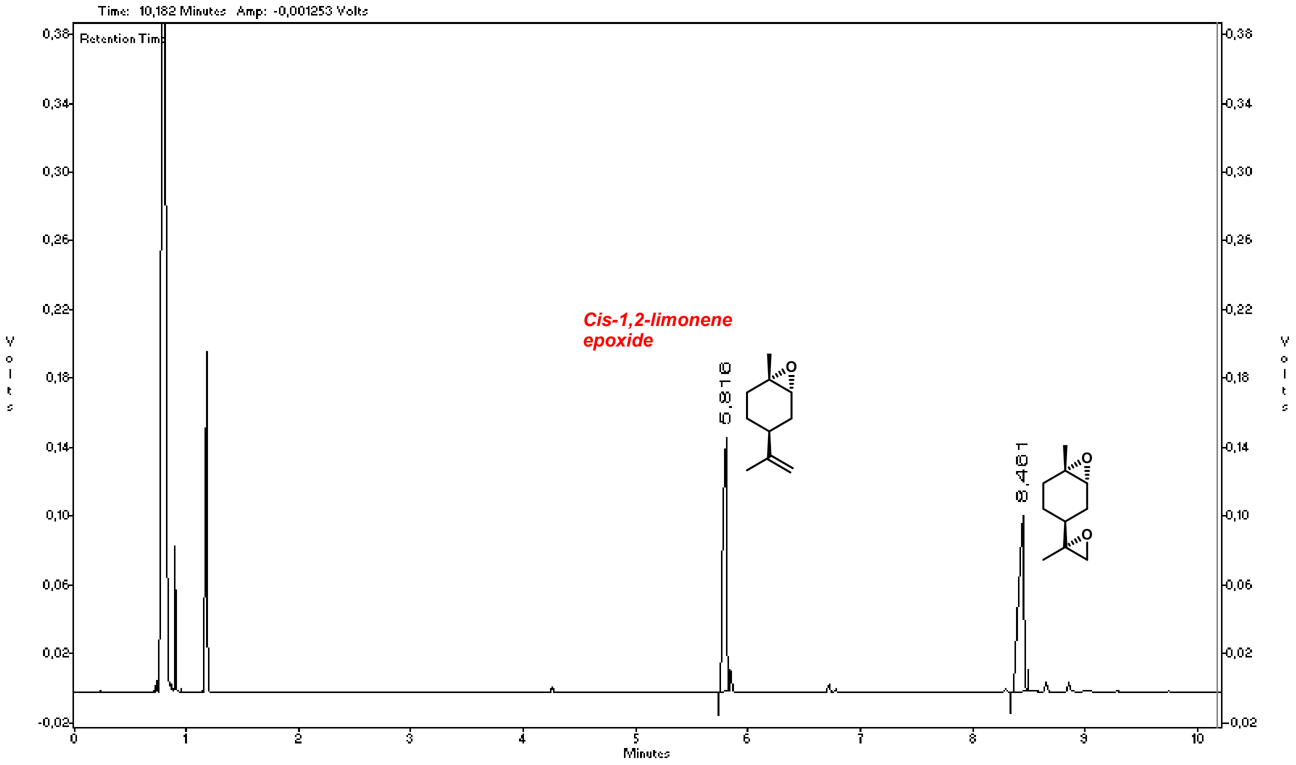

Figure S3. GC profile of the biocatalyzed epoxidation of *cis*-1,2-limonene epoxide from (*S*)-limonene to diepoxide at reaction time 2.5 h (about 50% conversion)

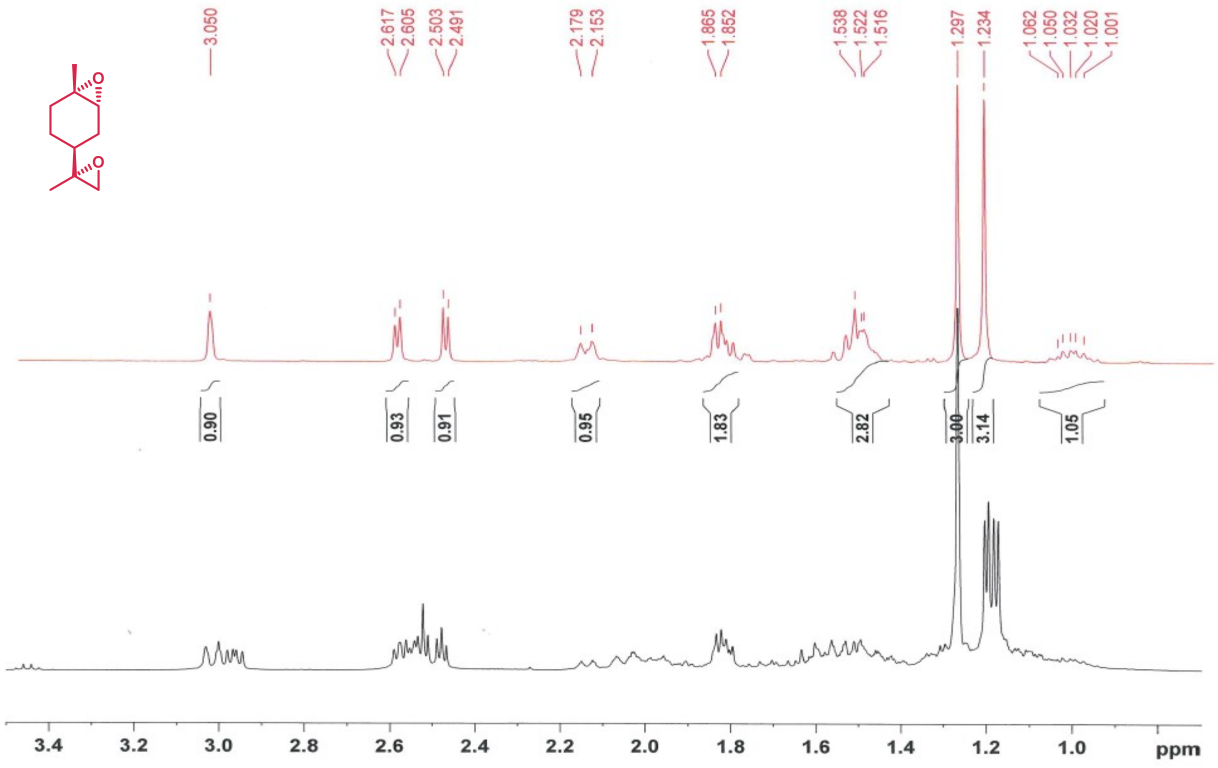

Figure S4. ^{1}H-NMR spectra of the mixture of diepoxides obtained by chemical epoxidation of (*S*)-limonene (black trace) and diepoxide obtained by biocatalyzed epoxidation of (*S*)-limonene (red trace)

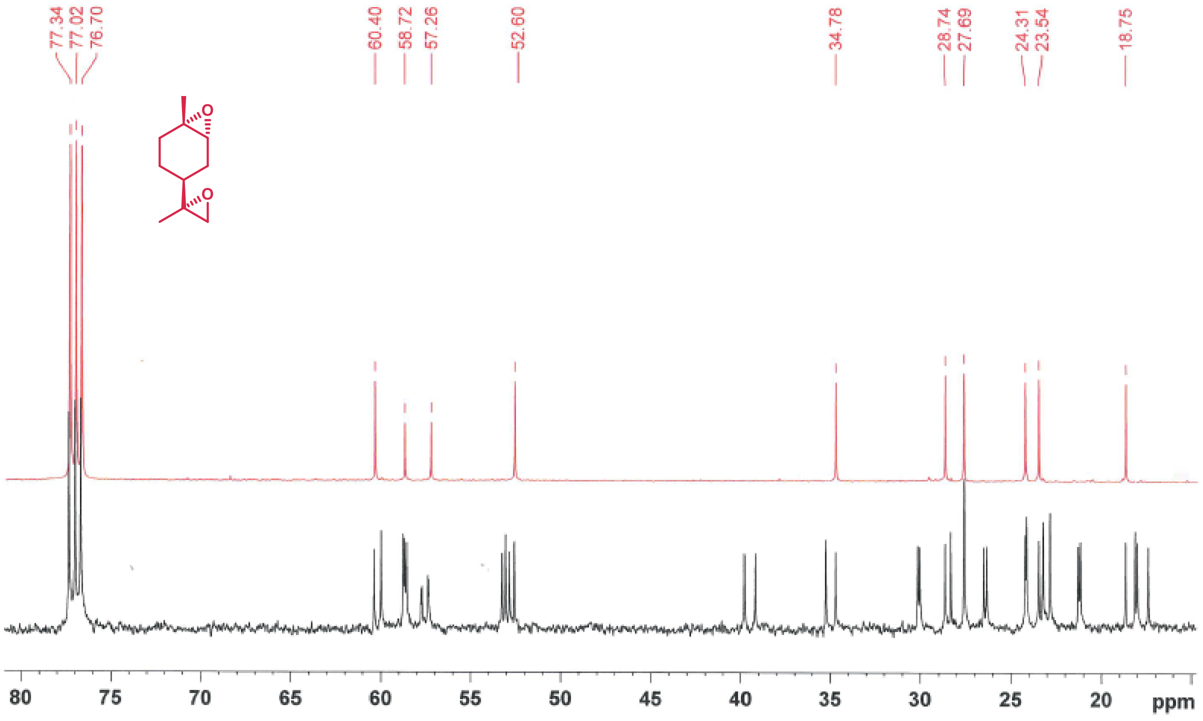

Figure S5. ^{13}C-NMR spectra of the mixture of diepoxides obtained by chemical epoxidation of (*S*)-limonene (black trace) and diepoxide obtained by biocatalyzed epoxidation of (*S*)-limonene (red trace)
